# The design and development of an experience measure for a peer community moderated forum in a digital mental health service

**DOI:** 10.3389/fdgth.2022.872404

**Published:** 2022-09-22

**Authors:** Charlotte Mindel, Lily Mainstone-Cotton, Santiago de Ossorno Garcia, Aaron Sefi, Georgia Sugarman, Louisa Salhi, Holly Brick, Katherine Jackson, Terry Hanley

**Affiliations:** ^1^ Kooth Plc, London, United Kingdom; ^2^ Department of Psychology, University of Exeter, Devon, United Kingdom; ^3^ School of Psychology, University of Kent, Canterbury, United Kingdom; ^4^ School of Environment, Education and Development, The University of Manchester, Manchester, United Kingdom

**Keywords:** digital mental health, online community, experience measures, multi-phased design, moderated forum

## Abstract

Online digital mental health communities can contribute to users' mental health positively and negatively. Yet the measurement of experience, outcomes and impact mechanisms relating to digital mental health communities is difficult to capture. In this paper we demonstrate the development of an online experience measure for a specific children and young people's community forum inside a digital mental health service. The development of the Peer Online Community Experience Measure (POCEM) is informed by a multi-phased design: (i) item reduction through Estimate-Talk-Estimate modified Delphi methods, (ii) user testing with think-aloud protocols and (iii) a pilot study within the digital service community to explore observational data within the platform. Experts in the field were consulted to help reduce the items in the pool and to check their theoretical coherence. User testing workshops helped to inform the usability appearance, wording, and purpose of the measure. Finally, the pilot results highlight completion rates, differences in scores for age and roles and “relate to others”, as the most frequent domain mechanism of support for this community. Outcomes frequently selected show the importance of certain aspects of the community, such as safety, connection, and non-judgment previously highlighted in the literature. Experience measures like this one could be used as indicators of active therapeutic engagement within the forum community and its content but further research is required to ascertain its acceptability and validity. Multi-phased approaches involving stakeholders and user-centred design activities enhances the development of digitally enabled measurement tools.

## 1. Introduction

Online peer communities can provide a platform of social interaction for young people. Children and young people are considered digital natives (1), most have lived with relative ease of access to the internet since childhood. This influences children and young people's attitude towards the internet and how they seek support, with most considering the internet as the first option for seeking information, advice, or emotional support for mental health problems (2, 3). When paired with the importance of social peers' attitudes, beliefs, and behaviour during adolescence (4) online peer communities can offer an important form of support for young people seeking information or struggling with mental health. Those online communities can be formed through instant communication tools, social media networks, and asynchronous forums, where people share content with the intention of being seen by peers.

The importance of online peer communities in supporting adolescent mental health is shown by a strong but complex relationship between online social networks, mental health and wellbeing (5, 6). When online social networks are used to seek support, reports of depressed moods are correspondingly minimised or maximised depending on whether users were passive or active in their online use (7, 8). However, some studies have reported low-quality connections, depression and “comparison effects” for specific social media platforms (9, 10). Liu and colleagues' (6) meta-analysis highlights how different digital communication tools and media usage affects wellbeing depending on the intimacy and activity type in the medium. More widely, the harm of some online social network platforms has been explored in more detail and found to predict an increase in body dissatisfaction (11). Therefore, it is important to recognise that the nature and characteristics of an online community will influence whether the impact on the mental health of users is positive or negative. For example, visiting pro-anorexia sites was negatively associated with perception of appearance, drive for thinness and perfectionism (12), whilst online social support has shown to act as both protective and risk factor mediating how web content is internalised (13). Conversely, online mental health communities can be seen as the analogue to traditional mental health face-to-face support groups, especially for a subset of the population seeking advice or to express emotions online (14, 15). The anonymity and social connectedness in these spaces can help people to overcome stigma and make positive disclosures of experiences and problems (16). Nevertheless, others have demonstrated how dependency on these communities can hinder recovery from stigma, especially in online spaces without moderation or supervision (17).

It is when online mental health communities have appropriate characteristics (e.g., moderation, anonymity, facilitation) that they can help individuals, and maximize support when experiencing mental health difficulties (18, 19). The potential negative impact of online mental health communities on young people can be mitigated using moderation of content, creating safety, preserving anonymity and other mechanisms to create a boundary environment which in turn is designed to avoid judgement and promote wellbeing. Observations of unmoderated platforms commonly identified signs of self-harm normalisation and increase of suicidal ideation (20, 21). Comparatively, users of moderated mental health forums report a reduction in frequency and severity of self-harm behaviour (22). Given the mixed impacts of online mental health communities, it is important to examine and attempt to measure experiences within these kinds of online communities, especially those designed to provide peer support, reduce risk of harm, preserve safety, and enhance wellbeing of online mental health experiences.

### 1.1. Measurement in online digital mental health communities

Determining how to evaluate the impact and effectiveness of online community mental health support should be a key focus for platforms providing online peer support services. The indirect and asynchronous nature of a community forum support presents challenges using standardised measures for its evaluation. This is particularly the case when the community is not directed to a target population, user-led, and not focused on a specific mental health concern leading to a specific outcome or mental health difficulty such as anxiety, eating difficulties or depression.

When standardised measures have been used in online communities' research, there have been mixed results. One online peer support group for young people found users improved in anxiety scores but did not show any changes in depression (23). Others found a non-significant reduction in depression of forum users or no differences in body dissatisfaction between forums users and the control group (24).

A clearer picture on the benefits of online mental health communities is found when qualitative and mixed methods are used. Horgan and colleagues (25) used thematic analysis on forum posts, alongside standardised outcomes for depression and anxiety. Young people using the forum frequently discussed the immediate benefits of sharing their feelings on the forum and described a sense of not being alone. Forum posts also mentioned the benefits of individuals comparing themselves to others, and consequently believing that their situation was less bad than previously thought. In regard to a self-harm community investigated, young people reported that they felt they learnt more about mental health from other users, compared to information sites, and they felt it easier to disclose information online, in part because they were less likely to be judged than in real life (26). More recent investigations have shown how self-efficacy and access to further support can increase thanks to the use of these online communities (27). They can also provide a sense of belonging, tackling feelings of loneliness regarding mental health experiences (28). Qualitative studies also reveal how social modelling allows encouragement between peers to use pro-social behaviour and receive support within and outside the community (29).

Qualitative methods do, however, have limitations in measuring outcomes for online communities at scale. They are time intensive and cannot be used repeatedly to track user experience and satisfaction, nor be used as a method to routinely collect information about the community. However, they provide an in-depth understanding of why young people use online mental health communities and what outcomes are achieved. The findings can be used as the functional theory to develop an experience measure for an online community.

Online peer mental health communities aiming to support users require understanding on how their resources and content help or hinder the user's wellbeing. Measurements can be collected and may be routinely aggregated to personalise a community experience in the platform or create automated recommendations of community resources likely to contribute to the recovery or support of the individual. Developing a self-reported measure for this endeavour should aid understanding of how helpful the content is, what kind of help the content can provide, and how different users may benefit from it. Ultimately, an experience measure will provide an indicator of therapeutic active engagement with the forum and community content, using a parameter of engagement that goes beyond the forum analytics and often reported digital contexts (e.g., Views, clicks, time, popularity). Measuring the helpfulness of community content may provide insights on how resources are consumed and contribute to a positive, negative, or neutral experience. The measure should also understand the mechanisms that lead to the helpful or unhelpful experiences in the community, and what types of outcomes users are achieving in relationship with their engagement with the forum content.

### 1.2. The peer online community forum within a Digital Mental Health Service

The Kooth.com (referred to from here onwards as Kooth) online community is a user-led forum inside a multi-component digital mental health service where the content revolves around the changing needs and experiences of the young people in its platform. The forum promotes a wide variety of professionally and non-professionally curated content about mental health and wider wellbeing topics, aiming to reduce stigma and contribute to meaningful conversations about people's mental health experiences. The content of the community consists of three core types of posts: (1) a co-created magazine with a combination of psycho-educational, creative, and informative content written by the service users and practitioners, (2) discussion forums authored by users, providing direct interactions between peers but still moderated by professionals (with mental health backgrounds), and (3) mini-activities, a specific type of content created with the intention of helping users build life skills and promote planned action. All user submitted content is moderated before being published on the platform to safeguard, categorise, and age-restrict content where necessary. The forum is part of a wider UK online service that provides with direct synchronous support with professionals, which is anonymous and free for users. When designing a measure for a specific online community and its characteristics, a framework to measure quality-of-care is required. These frameworks will be especially useful when the programme theory and mechanisms of change for the online support community have been previously investigated, so both can be combined to develop a specific and relevant measurement.

### 1.3. The Quality-of-Care measurement framework to develop a Peer Online Community Experience Measure

Donabedian's (30) quality of care framework recommends measuring care through assessing structure, process, and outcomes. For an online community forum, the quality of care is reflected in the structural elements of the community (e.g., Content, relationships, posts), the process or mechanisms of accessing support through peers and consuming content within the community, and the outcomes of the community that can be achieved when meaningfully engaged with it. Each of the three components have a bidirectional relationship. The structure of the community will influence the process of peer and community interactions, which will then impact the outcomes that are achieved. Positive or negative outcomes may change the process of peer interactions, and potentially change the type of content available within the structure. By using Donabedian's framework as the foundation for an experience measure, we seek to capture information into the forum community helpfulness (structure), peer and content interactions (process) and relevant reported outcomes for the individual.

The design, and structure of the Peer Online Community Experience Measure (POCEM) was divided into three parts, each representing one of the domains of Donabedian's measurement framework. The items in the measure were initially identified through the Kooth Theory of Change research where mechanisms of support and outcomes of peer support for the service were previously examined (31). Taking the framework approach adapted to the context of care relevant to the peer support community forum the measure is set up to assess the following:

a) **Structure assessment:** Assesses the quality of the community structure, focusing on “helpfulness” of magazine articles and forum discussions using a “emoji”-based Likert scale in response to the question “Did you find this part of Kooth helpful”.
b) **Process assessment:** Assesses why online community users found a specific structure (community resources) helpful or unhelpful, depending on their response to the helpfulness Likert rating. Respondents selected one of four support processes reflecting the possible interactions they were looking for in the community (1: Emotional interpersonal; 2: Emotional intra-personal; 3: Informational inter-personal; 4: Informational intra-personal).
c) **Outcome assessment:** Explores what outcomes are achieved, specific to the structures and processes considered helpful in reference to the area of support received. This means that a different subset of outcomes may be achieved depending on the mechanisms or processes of helpfulness that the user has previously identified in the assessments. Furthermore, within the context of Kooth there are two avenues of engagement for a community member, through active contribution in generating community content by writing, or through the consumption of content posted and available within the community by reading. These types of engagement are likely to be associated with different outcomes, depending on their role in the forum, whether the user is creating (contributor) or consuming community content (viewers). Therefore, the assessment of outcomes within the measure should be able differentiate depending on the user's role to inform the experience of the community as a whole.

A measurement that covers the three layers of assessment of quality of care should help to understand how peer support in an online community relates into a quality-of-care framework for the intended context, and how feasible is to measure the experience through an instrument tailored for a digital service context and program theory.

### 1.4. The present study

The present study describes the (i) development, (ii) user testing, and (iii) pilot results of the measure implemented in a dynamic and multifaceted digital mental health service. This study involved different key stakeholders and participants that influenced each phase iteratively. The ethos of Donabedian's framework (30, 32) was applied to the initial development of the Peer Online Community Experience Measure (POCEM) so the assessment domains of quality of care were included within the measure. In the (i) development subject matter experts and previous literature on the service were used aiming to answer the following question: *Which Items generated by subject matter experts best represent the high-level domain of support (process)?*

To ensure POCEM is a meaningful experience measure for the people using it, participatory think aloud protocols were conducted to guide the development of items and appearance of the measure with high-fidelity and clickable prototypes. Quasi-realistic simulations and (ii) user testing can help to discern the face validity of the instrument, and were used in this development process to address following research questions: *Does the respondent understand what is being measured? Do people understand the measure within the forum platform?*

Finally, to understand its feasibility as a measure, further observations through a (iii) pilot study to examine usage, completion, and item selection from the measure were collected within the online forum platform and mental health service, to answer the questions: *How acceptable by response rates is the phased measure within digital community? Do response rates influence scores for the instrument? And What are the most frequently selected processes domains and outcomes during the pilot?*

An iterative multi-phased design process aims to integrate evidence collected from practitioners, researchers, design experts, and young people. The design of the POCEM and its development provides an opportunity to collect data on the peer support community and assess structure, processes, and outcomes within the wider service for users. This study aims to provide a foundational design structure and outline a systematic development process for an online community measure, so others can be guided in the process to develop their own community experience measures, that are relevant and context specific, implications and lessons learned from each phase of the study are discussed. The study describes the mixed-method development of the POCEM divided by three phases including the implementation of the measure in a natural environment.

## 2. Methods

A multi-phased design process was used involving iterative development, reflective decision making and real-world application of the findings (33, 34). It is an iterative process that aims to integrate the perspectives of key stakeholders into the phases of measurement development into the digital domain. The development involved a group of practitioners, researchers, user experience designers as experts, young people from schools in which the service operates, and users accessing the digital forum community in the service. Their involvement as stakeholders and participants was iterative following three key phases of design, representing each study:

• *Phase 1, Item generation and reduction*: A three-part measure developed with digital product experts and designers. The content of the measure and items were created by combining qualitative thematic indicators of outcomes and mechanisms. Delphi rounds were used to reduce items and explore the content for the measure.
• *Phase 2, User testing*: To directly explore, using think-aloud workshops, the face validity of the measure with young people. The focus was to verify the appropriateness of language and how design of the measure was experienced on the platform as a prototype.
• *Phase 3, Pilot study*: A 10-week pilot of the measure within the digital online community. Exploring completion rates, average scores, item frequency selection and correlations between items scores.

**Figure 1:**
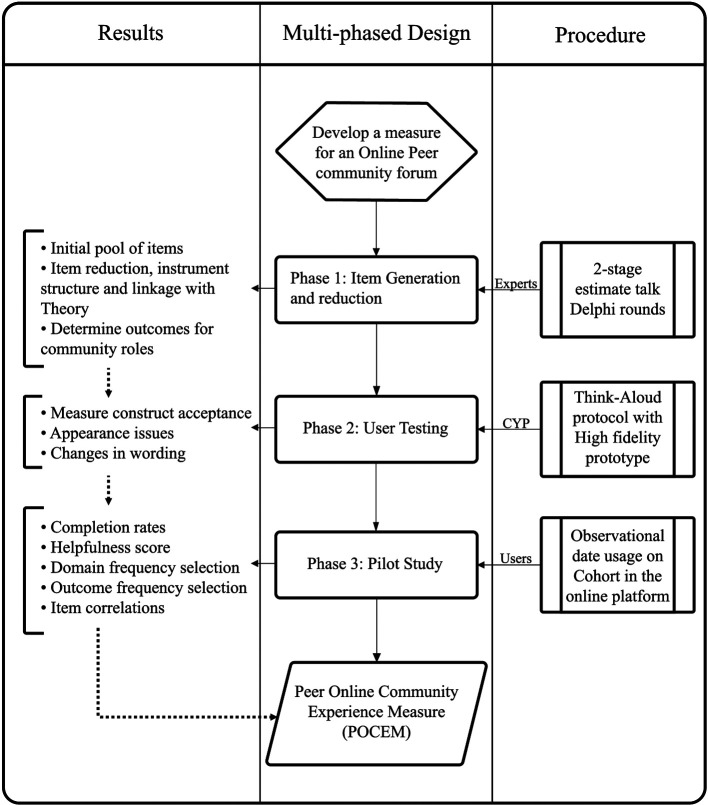
Multi-phased design with stakeholders and iterative results in the development of POCEM.

Each phase provides iterative results that inform the overall improvement and design and development of the measure, from theoretical foundation to practical design thinking decision-making. Multiple stakeholders and phases of development required a mixed-method approach with qualitative and quantitative data collection activities and incremental and iterative changes to the development of the POCEM. A break-down of stages, procedure, and results of the multiphase design is illustrated in Figure 1.

### 2.1. Phase 1: item generation and reduction

The process of item generation and reduction for the first phase of POCEM development was carried out using the Estimate-Talk-Estimate Delphi technique (35). The technique is used to achieve expert consensus through multiple discussion sessions between a panel of experts. The Estimate-Talk-Estimate method differs from the standard Delphi technique by then allowing for verbal interaction between panel members. The Delphi technique is frequently used in healthcare research and has previously been used to modify a social responsiveness scale (36), while the Estimate-Talk-Estimate method variation has been used in developing a framework for mental health apps (37).

To develop a measure of peer community experiences that reflected both young people's views and expert opinions, a two-stage Estimate-Talk-Estimate Delphi process was used. The first stage involved panel members with mental health practice expertise to compose an initial pool of items based on previous theory known about the service (31). The second stage of the Delphi process involved discussion between researchers to assess the items generated, identify links between the generated items and the constructs from the theory, and reduce initial pool of items using an inter-rater agreement approach to reach the final round.

#### 2.1.1. Participants

Two panel groups were recruited, with of a total of six expert panels for the Estimate-Talks-Estimate workshops. Most experts belonged to the service and one to a university institution. Emails advertising the research participation opportunity were sent to the service employees involved in research or in providing support to service users within the community, and to external researchers in the service's existing network. Experts registered interest in the project *via* email and specified whether they were interested in participating in (a) the service's Theory of Change thematic analysis, (b) the item generation stage of the online community measure, or (c) the item reduction stage of the online community measure. Experts could volunteer for multiple parts of the project. All panel members had extensive experience researching or providing support and moderation in the digital mental health platform.

Panel members were recruited to participate in two projects concurrently: the development of the service's Theory of Change (31), and the generation of the content for POCEM. One panel member was involved in both the item generation stage and the later item reduction stage. The continued involvement of one panel member was used to ensure continuity between the two stages (Table 1).

**Table 1:** Expert panel members for item generation.

Panel member	Organisation role	Institution	Project involvement
PM1	Mental health practitioner	Kooth	Thematic analysis; Item generation
PM2	Mental health community practitioner	Kooth^1^	Thematic analysis; Item generation
PM3	Lecturer in counselling psychology	University of Manchester	Thematic Analysis; Item generation; Item reduction
PM4	Lead researcher	Kooth	Item reduction
PM5	Chief research officer	Kooth	Item reduction
PM6	Lead experience designer	Kooth	Item reduction

**Table 2:** User testing—affinity diagram output from workshops with POCEM interactive prototype at Kooth.com.

Magazine	Discussion boards	General community experience	Measure Insights
Clearly seeing who authored content can build or break trust, impacting engagement levels	Comments are a powerful tool for support (if positive)	Seeing content that is positive, distraction based, or more generic life advice was well received and unexpected to first time users	Selecting multiple options would allow YP to explain more about why something was helpful
Relying on users to read and process large chunks of text is both off-putting and risky	YP will more likely respond accurately to content that relates to them or content that keeps them engaged	Peer support or “community” are important alternatives to counselling	Free text fields allow YP to add their own voice/explain themselves, which is important to feeling understood
YP want the ability to reflect on or re-engage with an activity or content that has previously helped or inspired them	Being able to explain themselves or detail the ‘why’ behind how they feel or feedback is important to YP	Moderation is an important safety net for YP and Kooth's policy is not made clear enough	YP may be more likely to respond to content that was helpful to them
		YP relate digital “social” styled interactions to social media	The measure was interpreted as being related ti the specific content or category
		There is some expectation that user feedback will result in more personalized site activity	It is helpful for YP to know who will see the results on what they interact with, as this can impact if they engage
		YP who may not be directly struggling are empathetic to others who may have nowhere to turn	

#### 2.1.2. Procedure

Four rounds of panel workshops were carried out with the expert participants. Workshops were held face-to-face and videoconference. Asynchronous communications through email were used to prepare the experts, and anonymous questionnaires for voting were provided in each round. The rounds had different aims regarding the content relevance and structure, reduction of proposed items and changes in wording of items to improve quality. All rounds were documented through field notes supervised by the research lead (TH).

##### 2.1.2.1. Initial round

The first stage of the process was a face-to-face meeting group with the expert panel, wherein a broad list of initial items was generated. The items were generated deductively using service programme theory (31). The experts involved in this initial item generation were concurrently involved in the Theory of Change research, allowing for a deeper understanding on the transcript's findings and theoretical foundation of the Kooth online community outcomes and mechanisms. The process of generating the initial pool of items utilized a thematic analysis approach, consistent with Braun and Clarke (38) analysis in psychology research. The thematic analysis investigated the factors influencing positive behaviour change for children and young people accessing an online peer support intervention and described the desirable outcomes for the online community. The items were generated by each panel member independently and decided in group through a panel discussion process. Items were generated using the framework for at least each of the desirable outcomes and mechanisms or processes for positive change in the online community identified in the thematic analysis.

##### 2.1.2.2. Item pool development rounds

**Figure 2:**
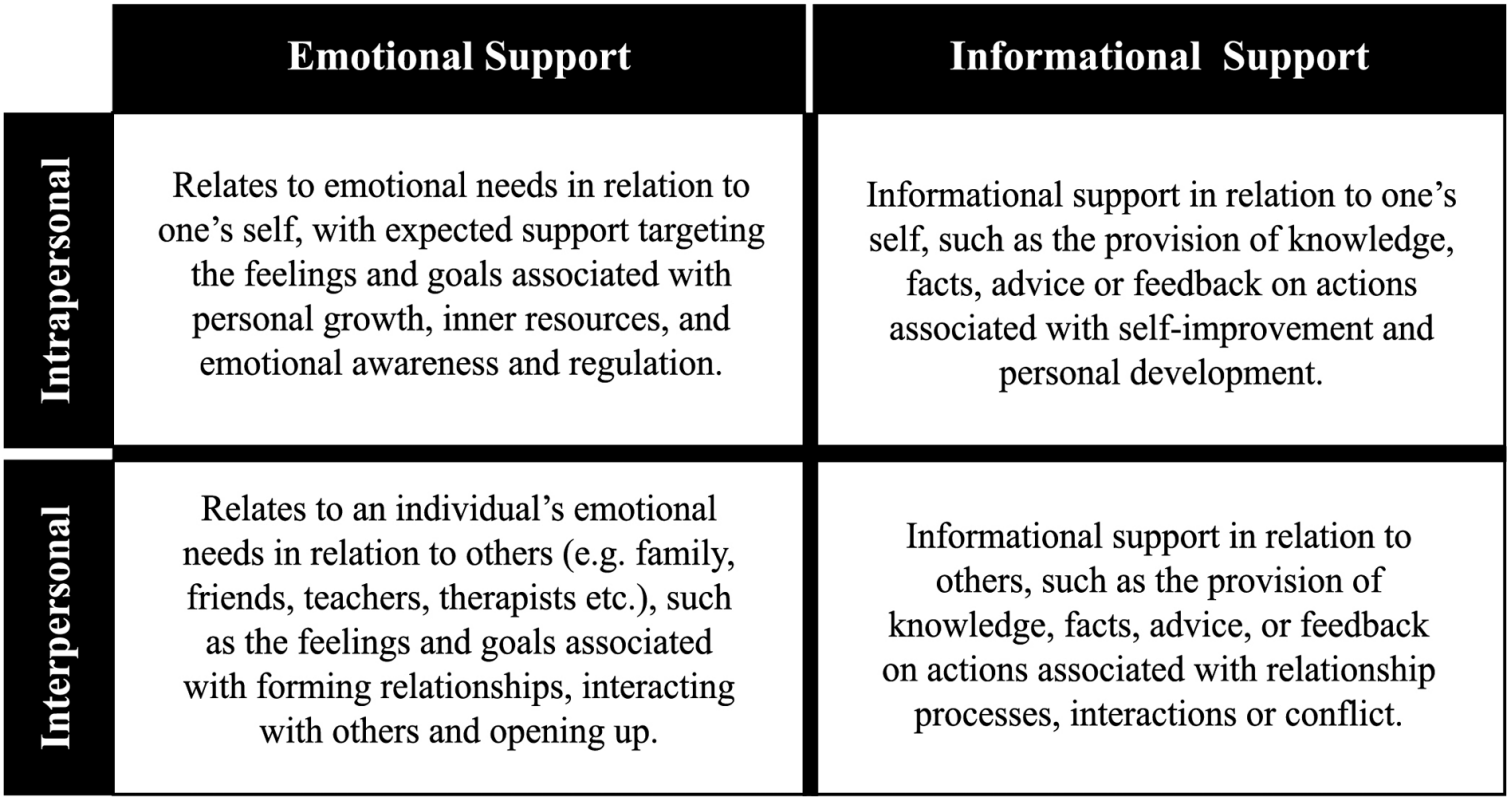
Kooth high-level domain of support (processes), “wants” and “needs” from children and young people accessing a digital mental health support service (31).

This round with panel members focused on mapping the initial pool of items to the domains of support. The domains of support were mechanisms formulated in earlier research, and they are intended to represent the high-level types of help from children and young people asking for mental health support within an online digital service. These domains of support are covered in the process assessment part of the POCEM, as the mechanism that leads to that online community resource outcome being helpful or unhelpful (Figure 2). After mapping domains for each item in the initial pool, panel members were asked to select the items more likely to be selected by two different types of online community members or roles: (i) those contributing; or (ii) those consuming (accessing by reading) the community resources. The workshop with experts focused on which items were more relevant to each type of community member and provided rationale on their decisions. The panel members voted on items' relevance to identify those to be discarded. Panel members were given two weeks to make their evaluations independently. A workshop was carried out to discuss the relevance items findings, and the relative ratings of the different members. The discussion focused on whether the items were repetitive, reflected the support domains as processes, and were representative of the outcomes from the thematic tree.

##### 2.1.2.3. Final round

In the final round, panel members were presented with those items selected through voting. Panel members were given three weeks to present their review. The workshop focused on editing the wording of the items, and further reducing the items down due to similarity, or coherence with the theory used to develop it. The discussion considered the independent comments made prior to the workshop to each item and comments recorded throughout all rounds.

#### 2.1.3. Analysis

Descriptive statistics were used to describe participants' characteristics and frequency in votes and selection was recorded for each expert. The field notes outputs from each round of the item generation and reduction phase were discussed sequentially, influencing the materials taken to the panel of experts in each round. In round three, when panel members independently and anonymously rated their preference of the items, an intraclass correlation coefficient (ICC) was calculated to understand agreement between panel members on their decisions for item selection.

**Figure 3:**
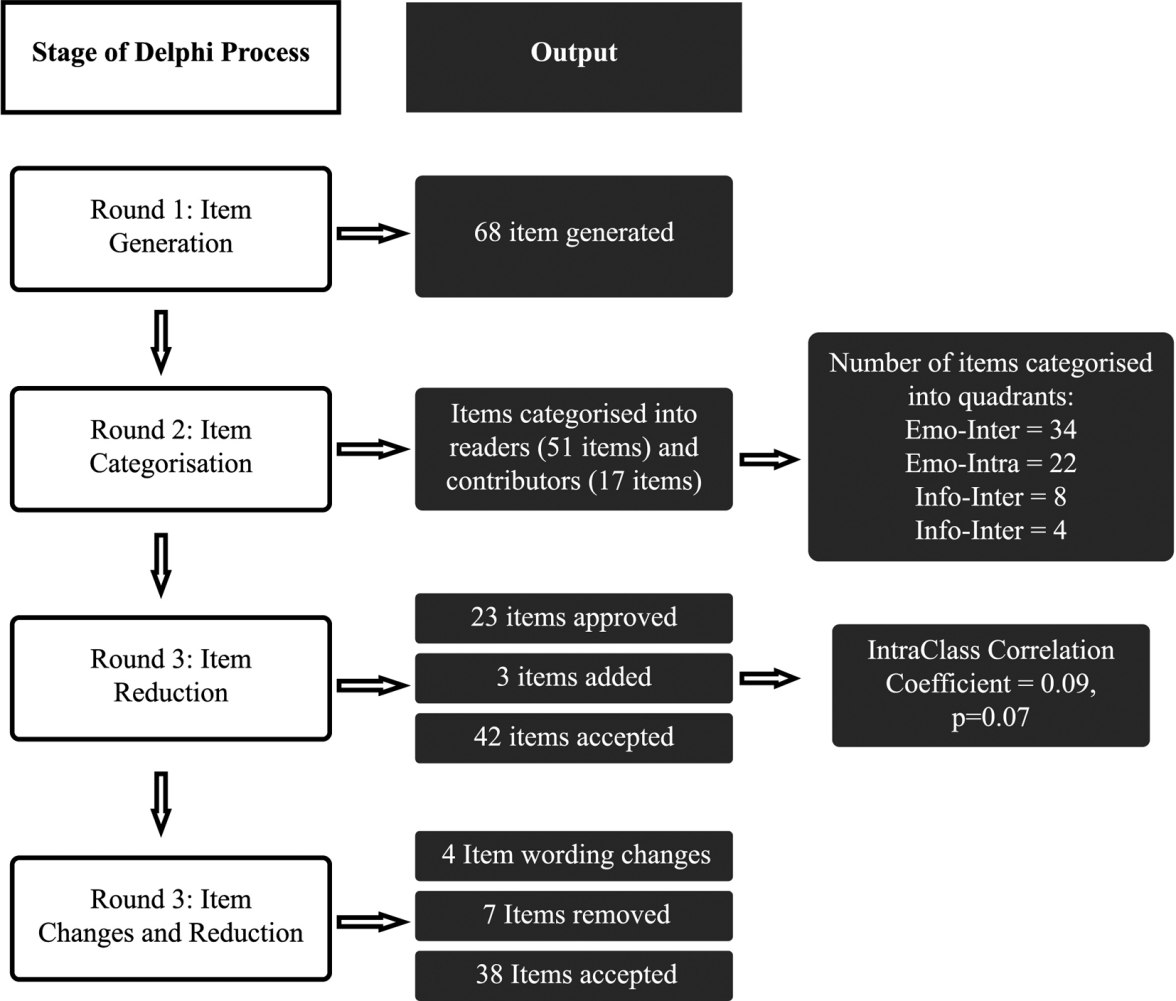
A flow chart of the results from each stage of the Delphi process for the generation and reduction of items.

The process of item generation and reduction was done iteratively over four Delphi rounds. A flow chart of the outcomes from each round of the item generation and reduction process are shown in Figure 3.

#### 2.1.4. Results

##### 2.1.4.1. Initial round

The items initially generated for the online community measure were produced based on the panel members' understanding of previous literature on online peer support communities and service's Theory of Change (31) describing a high-level domain matrix of the type of support that children and young people “Want” and “Need” from the digital service ecosystem.

The thematic analysis revealed desirable outcomes for positive behaviour change in the online community, the peer ecosystem: (i) Relatedness and Self-Expression; (ii) Hope and Help Seeking; (iii) Building a Safe Community; (iv) Digital Altruism; (v) Hope and Help Receiving; (vi) Making Change. The identified themes as outcomes were used to generate the initial pool of 68 items based in these desirable outcomes and categorised through their mechanisms or domains of support (see Supplementary Table SA1).

##### 2.1.4.2. Item pool development rounds

The first round aimed to divide items based on two criteria. First panel members categorized each item based on the type of community member that will find the item useful. Most of the items (75%; *n* = 51), were classified as relevant to users reading or consuming resources in the community, whilst the remaining 17 (25%) were relevant for users contributing with content to the community.

The second criteria was categorized into domains of support and it was used to inform the second part of POCEM. The emotional domains were more frequently used to categorize the items in the pool compared with informational domains (Table 3). Through a discussion process, participants agreed on a three-part structure to the measure, with respondents only shown items relevant to the selected process.

**Table 3:** Frequency of items categorization into high-level domains of support after round 1.

Wording of quadrant	Domain of high-level support	Frequency of items
“It helped me to relate to others”	Emotional-Interpersonal	34
“I feel better about myself”	Emotional-Intrapersonal	22
“I felt it was important to me”	Informational-Intrapersonal	8
“I learned some skills I can try with others”	Informational-Interpersonal	4

Each panel member voted on the items that they believed should be kept for the measure. An Intraclass correlation (ICC) estimate with 95% confidence intervals was calculated based on three judges, absolute agreement, with a 2-way mixed-effects model. The inter-rater reliability between panel members was poor at this stage of the item reduction process (ICC = 0.09, *p* = 0.07). In the following workshop the ratings were discussed amongst the panel. The outcome of the workshop was the removal of 23 items, and the addition of three further items. All the items that were selected by at least two ratters (12 items) were kept.

##### 2.1.4.3. Final round

Following independent and asynchronous evaluation of item wordings, nine suggestions were made regarding wording changes to the items. Six items were changed after the workshop where experts discussed ICC scores and disagreements between ratings and interpretation of the item (Table 4). The fourth and final round also resulted in the removal of seven further items. The output of the last round was a composed set of statements of three-part measure aiming to capture 38 different outcomes from four different processes on the helpfulness of community content and resource, this instrument content and structure was taken to prototype generation for the next User Testing phase.

**Table 4:** Changes made to the wording of items for the third part of the measure during the final round.

Original wording	Change in wording
I benefited from feedback from others	I benefited from comments from others
I feel optimistic for the future	I now feel more hopeful
I have implemented a suggestion from someone else	I have done something positive after a suggestion from someone else
I felt comfortable seeking support from my peers	I felt comfortable to share with others
I didn’t feel judged by others	I feel good about not being judged
I felt connected	I felt connected to someone

### 2.2. Phase 2: user testing

Once the development brought the content and design of the measure to a prototype, this was designed and integrated as a high-fidelity prototype using the current experience of the community forum. A further layer of validity is required when testing in digital environments, a human-computer interaction understanding to improve its face validity, but also to understand the overall performance and understanding of the measure by the target population, this should also improve construct and content validity with richer findings.

#### 2.2.1. Participants

A voluntary purposive sample of 11 young people was recruited amongst four primary and secondary schools in Manchester, UK. The sample was used to conduct user testing workshops. The 11 young people aged 12–17 (7 female, 4 male) expressed their interest for participating in the workshops. Young people aged below 10 or older than 18 were excluded from the workshops, as well as those at risk of safeguarding concerns or also not deemed appropriate by school staff to participate (e.g., lack of capacity or competency). The study was advertised through teaching staff, participants had no previous experience as users within the digital service (Kooth.com), parental and individual consent was sought for each participant and an incentive of £10 was given to participants to attend the 60-minutes workshops. Participants could drop-out of the workshop at any point. Two researchers, one with participatory research expertise and a user experience designer conducted and analysed the user testing sessions.

#### 2.2.2. Instruments and materials

##### 2.2.2.1. Kooth prototype: clickable high-fidelity

A high-fidelity prototype is a smartphone-based interactive representation of the product, the digital service, with strong similarities to the final design in terms of details and functionality. The high-fidelity prototype was developed with the vector graphic editor Sketch software (39) and included the POCEM inside the online peer support community allowing the users to click around and interact with the whole platform. In the context of measure usability, a high-fidelity prototype allows exploration of wording, structure, relevance, and comprehensiveness of the measurement and its functionality in interaction with the whole platform.

##### 2.2.2.2. Peer online community experience measure (POCEM)

**Figure 4:**
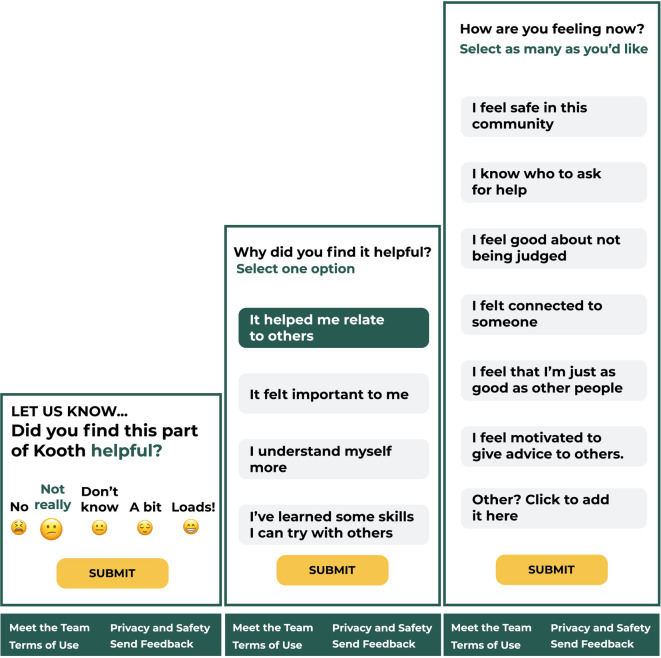
High-fidelity clickable prototype of the three stages for POCEM.

The POCEM is an online community measure designed and build on theory specific to the digital service (Kooth.com). Its aim is to measure areas of care around satisfaction and quality that an online community forum has in relationship with the individual in the context of the digital mental health service. The measure automatically differentiates between roles on the forum community by contributors and readers. The measure is divided in three stages and contains some logic based on the score responses and selection (see stages in Figure 4):

• The first stage contains single item question (“*Did you find this part of Kooth helpful?”*) scored with a 1–5 Likert scale (1: No; 2: Not really; 3: Don't Know; 4: A bit 5: Loads!; all scores aided with emojis) to assess the helpfulness of the online community resource. The Likert scale scores determines the helpfulness as a benchmark and branch the measurement into stage two.
• Depending on the scoring in stage one, a new single-item question will prompt “*What were you hoping for?*” for 1–2 scores and “*Why did you find it helpful?* for 4–5 scores for the respondents to select between four quadrants representing high-level domains of support from the service. Respondents who select score 3 “*Don’t Know*” do not complete more steps in the measure.
• The last stage is displayed only to those users who completed stage one and two (scoring 4–5 in step one and selecting the domain in step two), A single item (multiple response) question (“*What type of things have you learned?*”) will ask to select from a group of outcomes found at phase 1, readers can select between 23 outcomes and contributors can select 14 outcomes, the outcomes displayed will depend on the domain selected in the previous step, the outcomes available to select were generated in Phase 1 by experts.

##### 2.2.2.3. Lookback.io: screen recording / audio

Lookback.io (40) is a user testing software for mobile UX user recording tool. It allows recording of screen interactions alongside voice audio recordings when conducting supervised sessions of remote user testing (41). This software allows secure storage and organization of your user testing sessions for qualitative analysis. This tool allowed the recording of both screen behavior and audio from participants attending the user testing sessions.

#### 2.2.3. Procedure

The user testing was structured in one-to-one sessions delivered at each of the four schools. Participants were provided with a smartphone which had a loaded a high-fidelity prototype of the measure within the platform. Sessions were facilitated by a user experience expert researcher and observed by another researcher to safeguard the session and take notes. The sessions were voice recorded and screen recorded, for further transcript and analysis.

The facilitator encouraged young people to verbalise their thoughts and perceptions using the think-aloud protocol (42) as they navigated their way through the platform while following the facilitator instructions with the prototype. Instructions followed a protocol of specific tasks within the prototype measure that aimed to identify any issues with the interface, allowing facilitators to observed participant specific behaviour in relationship with the task. Facilitation tasks included asking about expectations in relationship to the next event that the interface showed during the session, and whether there were any issues with the wording clarity and relevance for the measure.

#### 2.2.4. Analysis

Affinity diagrams or KJ methods are adopted in user testing for prototype interaction (43). They are a good technique tool to synthetize and organize large amounts of qualitative data post-task, the user testing sessions were synthetized in affinity cards representing each participant's observations and quotes. Such cards are later jointly analysed to create a diagram. Affinity cards for issues more frequently raised, and for higher severity reported by participants tend to take more priority to address as changes in the prototype.

We followed the adapted four stage (creating notes, clustering notes, walking the wall, and documentation) process from Lucero (44). Researchers worked using rows to represent participants and columns for each affinity note. A total of 236 affinity cards were collected from field notes, screen recordings and audio recordings from each session. Rounds of clustering by researcher identified two main clusters in reference to the measure, and to the prototype and task performed (58.48% Measure affinity notes and 41.52% Prototype and tasks affinity notes). Twelve clustering issues were collected across clusters, some directly related with the measure such as including an “other” personalized option, and issues with the platform and prototype such as difficulties in navigation. The affinity diagram was then created, walking the wall exercises with other researchers and experts at the service (*n* = 3) provided with synthesis and identification of priority changes in the measure and prototype interaction by looking at frequency, feedback notes and quotes presented in the affinity diagram. Documentation on the output from the affinity diagram discussed by experts is provided in Table 2.

#### 2.2.5. Results

The affinity diagram findings identified issues and recommendations for the POCEM. Many participants perceived the measure to be linked with the type of content consumed or accessed-at-the-time by the user inside of the community, being mainly forum posts and subsequent comments.

This is well illustrated by one of the participants quotes when prompted in the session to explain what the measure is intending to do [Participant 3]: “*How? in my experience was just reading the person and the comments under it*”.

Most young people reported that they will be more likely to complete the measure if the content of the forum post was helpful for them.

The feedback suggests there may be an agreement bias effect deterring users from providing negative feedback to a peer within the online community, or encouraging users to ignore the measure when the content is not perceived helpful; [Participant 9] said “*If it is related to what I am doing, I would fill in the measure, to see similar articles*” and [Participant 1] stated that connection with peers will be a key motivation to complete the measure: “*… if I had trouble making friends I would say loads (of motivation)*”.

Most of participants found it normal that the measure will appear in piece of content within the community. Although, some participants expressed difficulty in finding the measure in the platform interface without prompts. This provided evidence regarding the measure appearance suggested that changes in design may affect measure completion. The comment from participant 9 indicated that some service users may believe that completing the measure contributes to content recommendation within the service. At the time of testing, content recommendation was not an intended outcome for the measure but highlights users' expectations and assumptions. It was identified that one emoji under the scale had a mismatched emotion. [Participant 10] explained: “The “No” just looks like they’re about to cry or something”. Despite the majority appeal to use emojis within the scale, for instance [Participant 1] stated his preference: “The emojis are more neutral not grumpy or red as might give wrong impression to others”. Findings also reveal difficulties from participants understanding who will see their responses. Four participants demonstrated doubts about the information being publicly available for peers to see in the community. For instance [Participant 8] showed: “I thought it would instil confidence in the author to write more”. User experience findings around physical appearance of the measure and its instructions led to changes for version of the measure taken pilot phase.

Finally, user testing workshops provided a scenario to review all item wordings based on experiences of participants interacting with the prototype during the exercises. Some statements changes are presented based on the rationale given by participants extracted from the affinity diagram. Wording review steered two changes on the process assessment part, and four wording changes in the last part of the measure (outcome assessment) (Table 5).

**Table 5:** User testing affinity diagram findings and changes in wording of the measure.

Original statement	POCEM	Rationale [Participant]	Changed statement
It feels important to me	Part two	“It means what he said is important to you because you relate to it” [N]	I learned something important to me
I understand myself more	Part two	“Maybe you don’t realise how you feel because maybe sometimes you can be sad but you don’t know why then you read all this it can make sense to you”[N]	I feel better about myself
I feel safe in this community	Part Three	“Does it mean Kooth as a whole or the outside community?”[N]	I feel safe in the Kooth community
I feel good about now being judged	Part Three	“At first I was like what does it mean?”[N]; “Obviously when someone judges you its negative, but in the app you can feel more better about not being judged”[N]	It feels good not to be judged
I have done something positive after a suggestion of somebody else	Part Three	“I don’t think you’re gonna do this—I don't get how you're gonna do something positive? I don’t think you’re gonna come back and do the quiz”	I have learnt enough to make a positive change
I feel excited to support other people with my new found knowledge	Part Three	“It is kind of the same as I’ve done something positive (Item) …”	Item removed

Overall, user testing allowed identification of appearance issues, validated the focus of construct measurement (it measures the specific community resource), and allowed changes on wording by the intended population. These findings allowed to implement and administer the POCEM measure at Kooth.com, providing the results of this implementation in the next face of the study.

### 2.3. Phase 3: pilot study

In contrast to content validity which is more concerned with having the breadth and accuracy of items to measure a construct, face validity assesses the degree of respondents judging that the instrument and its items are appropriate for the targeted assessment (45). For an experience measure to provide useful and valuable information, it must first be considered acceptable by service users within the context it is implemented. We used completion rates to assess how acceptable the measurement was within the online community and compared drop-out effects at each stage of the three-part assessment measure. The demographic differences were analysed between the assessment of structure scores, and the assessment of structure scores were compared between different outcomes and mechanisms. During a 10-week pilot we collected qualitative and quantitative data from the users in the online community completing the measure inside the platform (11–25-year-old service users).

#### 2.3.1. Participants

The measure was iteratively released onto the service's platform. Online service users who either contributed to a forum or submitted an article during the testing period were presented with the contributors' measure after submission. Service users who read an article or forum were presented with the readers measure at the end of the post. Users who did not provide research consent during sign-up to the service were excluded from analysis. Data was collected between the 13 November 2019 and the 22 January 2020.

#### 2.3.2. Procedure

The clickable prototype of the POCEM was implemented as a feature for service improvement in the online community at the service's platform, changes from the previous Phase 2 were included in the measure for pilot. For a period of 10 weeks the measure was tested within the platform and data was collected on users engaging with the community at the digital mental health service. Routinely collected monitoring information was used alongside peer support data to investigate the measure performance. All users accessing the platform community were able to complete and see the measure during the 10-week period.

#### 2.3.3. Analysis

Frequencies and descriptive analysis were carried out on completion rates for users who accessed the online community and those who completed the measure. Descriptive statistics and frequency of selection on the three steps of the measure were calculated to understand if items were being selected sufficiently. As POCEM measurement is divided in three assessments that interrelate, different analytical approaches were taken for each section of the measure. For the assessment of structure, the helpfulness score, Kruskal-Wallis non-parametric test alongside Dwass-Steel-Critchlow-Fligner pairwise comparisons post-hoc tests were used to ascertain differences in demographic variables (age, gender, ethnicity) on the score. Further analysis then explored the type of community interactions (whether the respondent was a reader or a contributor), using a two-sample *t*-test.

For the POCEM process assessment, we explored the effect of the domain selection on the score through Kruskal-Wallis, post-hoc analysis using Dwass-Steel-Critchlow-Fligner pairwise comparisons were performed looking at the average helpfulness scores for each four domains of support, and the average score for respondents who dropped-out at this step.

The POCEM outcome assessment was explored looking at the differences in scores based on the outcome selected in the measure. The aim of the pilot analysis was to see POCEM acceptability by service users using completion rates and whether the phased design resulted in a drop-off of respondents. We also explored the outcomes and processes more frequently selected by users of the measure while in the community.

#### 2.3.4. Results

##### 2.3.4.1. Completion rates

The measure was tested between the 11 of December 2019 and the 20 of January 2020, with 2,140 unique service users completing a total of 4,897 administrations POCEM. There was a total of 68,439 views of community content on the site by service users who gave research consent during this time, and a total of 2,425 contributions in the form of article or discussion posts in the online forum community. Completions rates were divided between readers and contributors to better understand overall completion of the instrument across community members (Table 6).

**Table 6:** The unique users, POCEM completions, and proportion of completions within the community.

Engagement type	Participants	Frequency of measure views	POCEM total completions	Community completion rates
Readers	2,083	68,439	4,685	6.85%
Contributors	57	2,425	212	8.74%
All	2,140	70,864	4,897	6.91%

##### 2.3.4.2. Participant demographic characteristics

The respondents ages ranged from 10 to 25, the range allowed in the community and the service. However, five respondents reported an age over 25 and were removed from the dataset, as these will be outliers of the service. The remaining respondents ages ranged from 10 to 25, with a mean of 13.47 (SD = 2.09). Most service users completing the POCEM were female, white, and aged between 10 and 14 years (Table 7).

**Table 7:** Demographic characteristics and frequencies of unique users completing POCEMs.

Demographic	Frequency	Relative frequency
Gender		
Female	1562	73%
Male	474	22.2%
Gender Fluid	71	3.3%
Agender	33	1.5%
Age		
10–14	1143	72.6%
15–19	557	26.0%
20–25	46	1.4%
Ethnicity		
Asian	128	6%
Black	81	3.8%
Mixed	109	5.1%
White	1753	81.9%
Other	69	3.2%

##### 2.3.4.3. POCEM structure assessment: helpfulness of peer community

The most frequently selected helpfulness score was 5: “Loads!”, indicating that the content helped the service user considerably. The frequency with the rating of 1: “Not really” was selected the least frequently. The mean helpfulness score was 3.77 (SD = 1.14).

Demographic differences were analysed to investigate whether the POCEM showed different experiences of structure between service users.

There were no significant differences between different genders [*H*(3) = 2.4, *p *= .40], or between different ethnicities [*H*(4) = 8.4, *p *= .07]. The age had a small significant effect on the perceived helpfulness scores, with a Kruskal-Wallis test showing a significant effect of age on helpfulness score [*H*(2) = 7.89, *p *= .02]. Post-hoc tests using Dwass-Steel-Critchlow-Fligner pairwise comparisons were carried out for three groups and showed service users aged 10–14 gave a significantly (*p *= .03) higher helpfulness score (*M *= 3.8, *SD *= 1.13) compared to service users aged 15–19 (*M *= 3.7, *SD *= 1.5). There was no difference (*p* = 0.4) between service users aged 10–14 and aged 20–25 (*M *= 3.4, *SD*= 1.43), or between service users aged 15–19 (*M *= 3.68, *SD *= 1.15) and aged 20–15 (*p *= .80).

**Figure 5:**
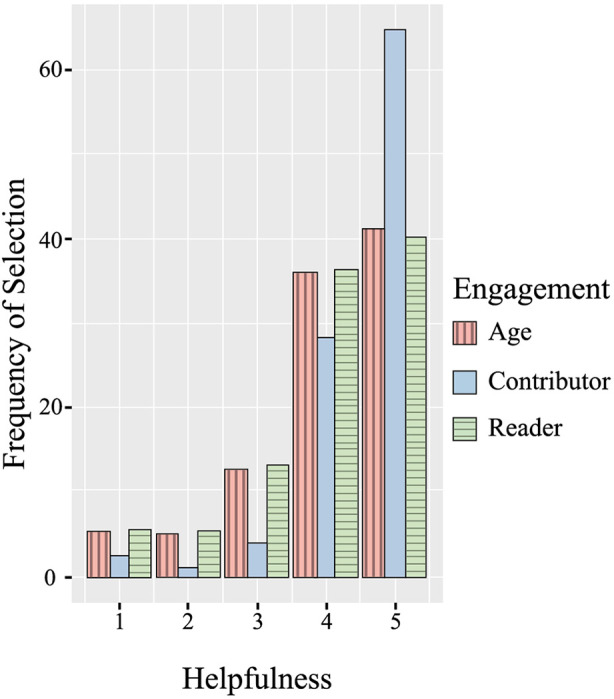
Frequency of selection across the five helpfulness scores for each type of engagement.

For the role as a member of the community, T-test frequency comparisons showed a statistically significant difference in the mean helpfulness score [*t*(247) = 8.8, *p *< .001] between readers and contributors. Service users who completed the POCEM after contributing to the community content selected the helpfulness score of 5:'Loads!' substantially more frequently than service users who read the community content (Figure 5).

##### 2.3.4.4. POCEM process assessment: high-level domain of support selection

Out of the 4,897 completions of the measure, 14.2% of responses gave 3: “Don’t know” as the helpfulness score. For this score response, the rest of the measure was not shown, and responses (*n *= 619) were removed.

**Figure 6:**
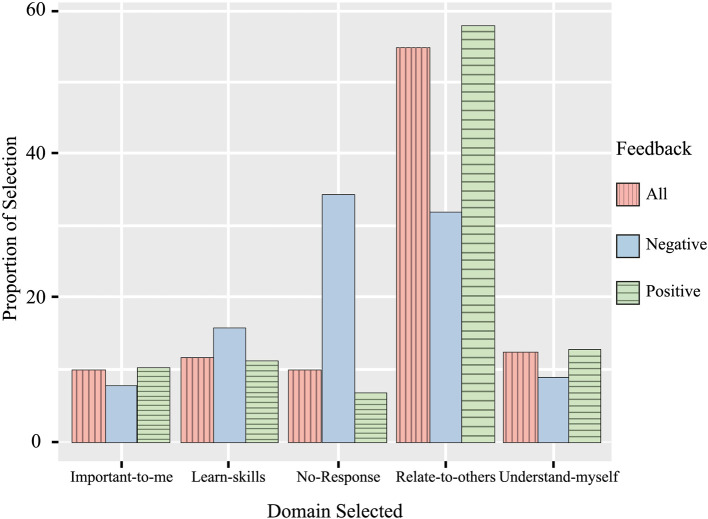
Frequency of selection for high-level support domains in process assessment of the measure for each type of feedback.

As seen in Figure 6, the most frequently selected high-level domain of support was “Help me relate to others”, with 55.1% of respondents selecting the option. Across respondents who gave positive feedback more than half (58.2%) selected the domain from the process assessment “Help me relate to others”. When looking at respondents who gave negative feedback, 32.3% selected the “Help me relate to others” (Emotional-Interpersonal) domain of support.

Out of the 4,278 responses with a score positive or negative score (1,2,4, or 5), 10.05% of respondents stopped answering the measure after providing a helpfulness score. When splitting the responses by positive or negative feedback, 34.5% of those giving negative scores did not answer next process assessment part of the measure and dropped out. Comparatively, out of all respondents who gave a positive response, only 6.8% dropped out of the measure without selecting a process domain.

An analysis of the helpfulness scores for process support domains gives the same message as the frequency findings, with respondents who dropped out of the measure giving a lower average score. A Kruskal-Wallis test showed a significant effect of process domain selection on helpfulness score [*H*(4) = 207.45, *p *< .001]. Post-hoc tests reveal that there was no significant difference between the helpfulness scores on the domains of POCEM process assessment. The post-hoc analysis revealed more about this difference and showed service users who did not give a response (“No response”) gave a significantly lower helpfulness score (*m* = 3.28), compared to those who selected the other domains “Important to me” (*M *= 4.29, *p *< .001), “Learn skills” (*M* = 4.09, *p *< .001), “Relate to others” (*M* = 4.31 *p *< .001), and “Understand myself” (*M *= 4.28, *p *< .001) (Table 8).

**Table 8:** Dwass-Steel-Critchlow-Fligner pairwise comparisons for domain helpfulness scores.

Item 1	Item 2	W	*p*
Relate-to-others	No Response	−19.784	<.001
Relate-to-others	Learn-skills	−1.855	.684
Relate-to-others	Understand-myself	1.991	.623
Relate-to-others	Important-to-me	2.278	.491
No Response	Learn-skills	12.733	<.001
No Response	Understand-myself	15.671	<.001
No Response	Important-to-me	15.011	<.001
Learn-skills	Understand-myself	2.683	.319
Learn-skills	Important-to-me	2.889	.246
Understand-myself	Important-to-me	0.397	.999

##### 2.3.4.5. POCEM outcome assessment: outcome based selection

**Figure 7:**
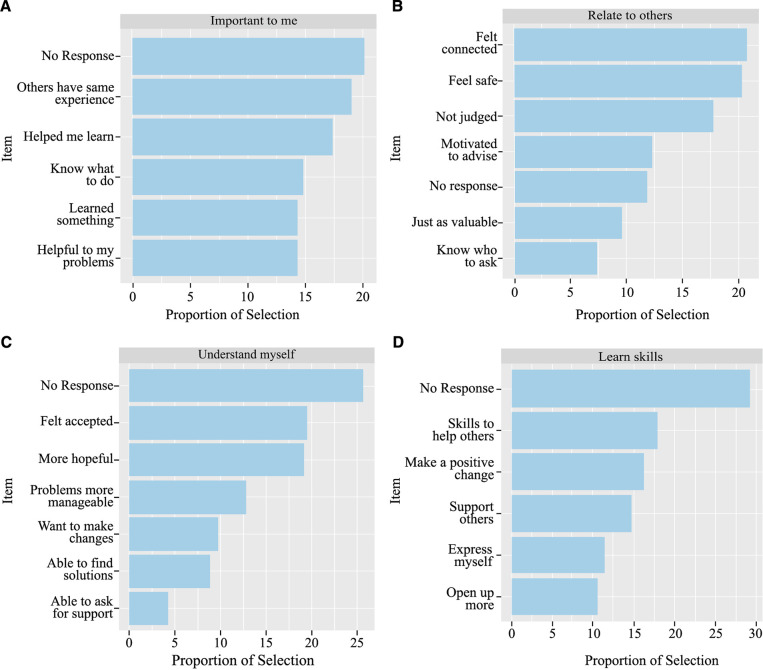
POCEM selection frequency of outcomes for each domain. Each panel shows the proportion of selection for outcomes selected by High-level domain of support selection: (**A**) “Important to me”; (**B**) “Relate to others”; (**C**) “Understand myself”; (**D**) “Learn skills”.

The analysis was run after removing cases where respondents did not answer the process domain selection phase (*n *= 430) as drop-out no scores were recorded in the administration. The frequency of outcome selection was analysed for each of the process domains, as the outcome items shown to respondents was dependent on the earlier selection. For all but “Relate to others”, the most frequent action from respondents was to drop-out of the measure, making up 20% or more of the responses in each domain (see Figures 7, graphs A–D). When looking across all the outcome responses, dropping out of the measure after the process question accounted for 25.38% of the sample who reached this stage of the POCEM. This is a higher drop-out rate compared to the 10.05% of respondents who dropped out at the previous stage.

For the domain “Important to me” the outcome item selected most frequently was “Others have the same experience” (18.9%), for the domain “Understand myself” the item “Felt accepted” was most selected (19.5%), and for the “Learn Skills” domain the most selected item was “Skills to help others” (17.9%). The process domain “Related to others” had the item “Felt connection” selected the most frequently (20.7%) (Figure 7, graph B). The item “I now feel able to ask for support outside of Kooth” was selected the least frequently out of the total items, with only 4.27% of respondents selecting the “Emotional-Intrapersonal” domain choosing the outcome (Figure 7, graph C).

The pattern of lower helpfulness scores for respondents who dropped out of the measure before completion continued for the outcome item stage. The Kruskal-Wallis test showed a statistically significant difference in helpfulness score between outcomes [*H*(22) = 407, *p *< .001], and the post-hoc test showed a significant difference in the helpfulness score when respondents dropped out before answering the outcome assessment stage, compared to those selecting an outcome in the instrument. There were no significant differences between other selection of outcomes (Supplementary Table SA2).

## 3. Discussion

This study outlines and discusses a novel multi-phased design method for developing an experience measure for young people within an online mental health peer community forum. We aim to provide a structure for design to improve experience of technology-enabled solutions in an online mental health service, whilst reflecting on lessons learnt, to support the future research of experience in other digital mental health contexts. We highlight the value of using mixed methods for an iterative design process with structured phases of data collection and synthesis.

Previous research has shown that role of online peer communities in supporting mental health is complex, and not always positive. There is a clear need for digital services to monitor the experience of service users engaging with online communities when the community is offered as part of digital mental health support, to understand whether the community is truly effective at providing mental health support as part of the service. An experience measure can provide an evaluation of the quality of care received by service users, uncovering what is and it is not working in a service (46). Using the Donabedian framework (30, 32) as the framework for measure design enabled an assessment of how the structure, processes, and outcomes within a specific mental health online community are experienced by young people and provided a theory-driven model for measure creation.

We conducted a phased approach for instrument development involving research with multiple stakeholders and mixed-method data collection activities, implemented in a real applied context (Kooth.com). A phased approach can help the implementation of the instrument by two processes: (1) design and (2) evaluation, of each phase iteratively and gradually optimizing the solution for the technology-enabled service, to ultimately sustain it (47). The multi-phased design was structured in three study phases to answer specific research questions relevant to stages of measurement creation (48). First, experts to design the principles of the instrument, providing the foundation for a prototype. Second, young people as participants of user testing think-aloud protocols provided feedback on what they considered important to measure and their perceptions interacting with the measure as a prototype. Third, we evaluated in a pilot study the usage and completion of the measure within the platform with users as stakeholders.

The phased design involved multiple stakeholders, but each contributed to a singular phase. In the development of the measure itself, young people were consulted (phase 2), with much of their feedback influencing the final design. Involving young people across the development and design was essential for ensuring the instrument accurately reflected their needs within a peer online community, and therefore improved its acceptability (49). A phased approach for instrument development involving co-design participatory action research with multiple stakeholders can influence the structure and purpose of the measurement. Similar approaches are useful to influence the government policy on digital mental health in Australia (50). In the case of using community-led design for the development of an experience measure, findings and design decisions may be counterintuitive to the structure and administration of the instrument. For example, the design and administration may limit or breach assumptions to test measurement performance in psychological instrument research enhancing difficulties to understand the psychometric properties of the measure. On the other hand, ensuring the contribution of user stakeholders is embedded in the development increases the likelihood of acceptability and adoption within the given context. A consequence of placing service users, clinicians, and user experience experts at the centre of the design process may be an atypical structure to the measure or solution with competing the needs reflected in the process and the final solution and can add some complexity to the development process. This uncommon structure may hinder its generalizability and may not adhere to assumptions required to further investigate the quality of a measurement and its validity.

As a lesson learnt, it is important to carefully understand in which phases each stakeholder should influence and consider a wider involvement with participatory roles within each multi-phase design. This in turn may influence the time and complexity of this type of implementation in a technology-enabled service, as more complex data and synthesis will be involved.

This phased approach using the context-specific theory provided by the Theory of Change of the technology-enabled service (31), which should increase adoption and success on acceptability providing high response rates. Despite this, the pilot results found low completions rates, and a high drop-out rate as the stages of the measure progressed. Similar studies focusing on theory-driven instrument development found low response when surveying different services (51). This brings wider questions on how online communities and their mechanisms and outcomes should be investigated and subsequently measured. Future research aiming to refine the experience measure development process should consider how variations in the measure structure may impact acceptability by service users, alongside the transferability of the measure and the phased-design process to other communities and their own theory-driven frameworks.

Regarding instrument development, the first phase of the process focused in answering whether the theory-driven items generated by subject matter experts sufficiently represented the domains of support (also considered processes). The use of adapted Estimate-Talk Delphi rounds with experts allowed for a narrowed and improved content of the measurement. The approach was non-standard, with each Delphi round composed of two parts; an initial, independent assessment of the items followed by a group workshop. Unlike systematic approaches to Delphi rounds (52) the independent and anonymous polling incorporated both qualitative and quantitative feedback. Whilst most Delphi rounds included independent voting, only one round required panel members to rate the items. In the other rounds, panel members provided feedback only. Although rounds may have benefited from a psychometric systematic assessment like Content Validity Indexes (53), a dynamic approach to the Delphi provided rich qualitative feedback to influence the iterative design of the measure and enhance the design of the innovation (54). Feedback in the initial Delphi rounds suggested the three-part structure, with the helpfulness rating placed at the beginning, filtering the other parts of the instrument collecting information about mechanisms and outcomes of that community experience. The theory-driven items were carefully selected through this process and deemed relevant for the context, and a consensus was reached to directly map the items onto the processes of support identified in the Delphi rounds.

However, the three-part structure presented challenges on quality. The drop-out rates found in the pilot testing suggests that the measure structure was not adopted by service users. Whilst in many context-specific measure creations, experts will be at the core of the design and creation, researchers should sense check design by the target community, with key stakeholders consulted at multiple stages of the development process.

The User testing phase was considered by the research team a fundamental step for measure development in an online context, it aimed to acquire face validity by asking young people about their understanding of what is being measured, and if people understand the measurement within the technology-enabled service. We recommend replicating the best fidelity prototype possible when conducting user testing research activities, despite low and high-fidelity prototypes have shown similar results when compared (55). We observed how young people benefited from structured activities and more realistic objects for the think-aloud exercises, which in turn can help to influence changes in the appearance and quality of the workshop outputs and its findings. User testing is a time-consuming process and the volume and complexity of data generated may contribute to longer periods of time and expertise needed for analysis. The KJ method can provide a good opportunity to analyse and synthesize findings but requires expertise and focus from researchers to facilitate the synthesis of the user testing activities. Affinity maps on the other hand can inform beyond the purpose of the research and provide ideas and improvements with general industrial value (e.g. user needs, product satisfaction). The findings from the user testing indicated that the participant understanding of what was being measured matched with the goal of the measure (to measure the experience interacting with content in the community).

In product development, usability research focuses on identifying areas where users struggle with a product or start to lose interest through observing people interacting with the product whilst trying to accomplish goals or tasks (56). For POCEM the participants were prompted to complete task but allowed exploration to gather their thoughts and cognitions about the wording and understanding of its purpose. This phase is likely to influence the item development process and provide further evidence on content validity of measurements in digital contexts.

User testing will often use a volunteer or purposive sample, added emphasis should be placed on finding participants normally underrepresented, as well as ensuring safety and ethical standards for research with vulnerable populations. User testing methods may present challenges integrating quantitative information like usability surveys, but this may help to improve researcher bias in the synthesis stage. User testing represents a new and additional phase for measure development that provides invaluable observations about the digital context and measure content (57). As wording of items were influenced by this phase findings, other studies should consider involving the target population in the initial item and theory development using community-based participatory research approaches (58).

Our pilot study set up to understand how acceptable the measure and how acceptability bias may influence scores. Results indicated that service users who completed the measure during the observational period had a positive experience when accessing the online peer community content, with the helpfulness ratings frequently positive. Those who contributed to the community as writers found it more helpful than those who consume the community as viewers, whilst younger service users rated the experience in general as more helpful. Given that most of the service users are aged between 10 and 14 years (72.6%), a lot of the content both written by service users and by the Kooth content team will be targeted towards younger service users. As such, it is not surprising the young service users may have a more positive experience within the community.

These initial results may indicate social desirability or acquiescence bias effects, previously found in digital contexts and scale creation (59, 60). The potential influence of an agreement bias effect was highlighted in the user testing phase, the majority of young people interviewed by think-aloud protocols reported that they were more likely to complete the measure if the specific content of the forum was considered helpful. Four out of the 11 participants indicated that they believed providing feedback would automatically notify the author contributor of the forum post. The user testing phase revealed worry from users about their responses being seen by other community members, changes in the instructions and text in the measure were applied in the pilot phase, including further instructions reinforcing anonymity of responses (Figure 3). The limited disclosure required to complete POCEM responses, along with the anonymity of the service, should help users to not anticipate a social consequence of their responses (61, 62) and promote completion and engagement with the measure.

The completion rates for POCEM during the study were low compared with the potential size of the community represented by the number of views during the pilot. Furthermore, from those who started the measure a respondent fatigue effect in each step of the measure was observed. The low competition rates and high drop-out rate of service users starting the measure presents a key challenge and threat to the acceptability of the measure. Users reading content in online communities are more likely to be “lurkers”, individuals who will read community content but not actively participate (63). Therefore, digital environments might be more prone to missing or misleading data after administration, how missing data is treated can have consequences in psychometric testing and measure performance (64). Researchers in digital contexts should be aware of these issues, report missing data or exclusion rationale, and think in advance what psychometric properties or indicators of quality for the measure should be tested.

In regard to the frequency of selection of domains and outcomes, the pilot showed support domain “Relate to others” to be the most frequently selected process for those service users who perceived the resources as positive in terms of “helpfulness”. The average helpfulness score for users selecting the domain was not significantly different to the other support domains, but service users were less likely to drop-out at the item selection stage when selecting this domain. Previous research has illustrated key reasons for young people seeking out support in online communities are to feel less alone with their problems, find a space to talk with peers, and find a space where they feel less likely to be judged (26). Our pilot results show similar reasons for young people seeking support, with the most frequently selected outcomes in POCEM for positive experiences in the community being “Felt connection” and “Felt accepted”. Similar outcomes for a supportive online community have been found previously (65) and demonstrate how online communities may help users to feel less isolated and more supported (66). The overall frequency of outcome selection at the outcome assessment part of POCEM will, at least partially, be a consequence of the differences in frequency selection of domains in the previous assessment. When respondents selected an outcome after selecting a domain in the process assessment, there was no significant difference between the average helpful scores for each outcome. The positive average of helpfulness during the pilot reflects a positive experience for service users in relationship to the outcomes selected in the instrument. On the other hand, it may also be a consequence of a ceiling effect in the measure (67).

There are several limitations to be considered for the development of the POCEM. This study offers insights into considerations that should be made in the early development of a measure for a digital context. By designing the instrument or measure with a specific service in mind, the ability to generalize the existing measure to other online peer communities is limited, and the use of experts from the same context may provide a limited view during Delphi rounds (68). Some of the lessons learnt in the development of the POCEM illustrate the benefits and challenges of designing and testing a measure in a digital environment within a multi-phased mixed methods approach. Further statistical and content validity testing is required, especially to understand how individual differences may affect the performance of the measure, and if biases of its design can be reduced by optimization cycles.

The POCEM can help us understand consumption and use of mental health supportive online communities beyond web-based analytics (e.g., how long people read, or contribute, and how frequently they engage). Further exploration on acceptability and completion rates in relationship with other digital phenotypes and instruments are required. Routinely collected information from this measure may help to understand the trends and commonalities deemed helpful in the community, it should also explore differences across population characteristics so the measure can be evaluated beyond the pilot, thus one should be mindful of the demographic differences observed and how they may affect future applications of the measurement and biases. If the measure is found in future research to have a sufficient level of acceptability by service users, then a further direction of research is to investigate the use of the measure by informing content recommendations. In the user testing phase, one participant believed that filling out the measure would result in personalised recommendations, suggesting that a personalisation experience may be expected or desired by service users.

Experience measures like POCEM can help services to understand the mechanisms and outcomes more frequently achieved by users of an online community. Online peer communities may use experience measures to understand what resources benefit or hinder the individual, so “active therapeutic engagement” can be monitored and better understood beyond digital analytics, so a positive and safe space and ecosystem can be maintained for peer support in a digital mental health community. Research aiming to replicate the development process for an experience measure in a different context should consider whether a greater level of involvement from service users could improve the acceptability and completion rates, as sustainability and adoption within the technology-enabled service will be more likely to be achieved.

## 4. Conclusions

Developing an experience measure for an online community requires a multi-phased systematic process, its development should be informed and structured involving stakeholders. Different stakeholders can contribute to pieces of information leading to key decisions on the design and development of the instrument. A phased approach with multiple methodologies and careful selection of stakeholders for the appropriate time and stage of measure development is recommended. Delphi expert rounds and think-aloud protocols provide rich data that can influence the structure and construct validity of the measure. Further studies are needed to understand psychosocial factors and causal explanations for supportive online communities' outcomes, particularly outcomes related to mental health and wellbeing. Measurement of self-reported helpfulness or experience of community content may serve as an indicator for “therapeutic active engagement” in a digital service and help to understand the main reasons users benefit from these communities and its content, but further psychometric testing and evaluation is required. The pilot findings collected on outcomes is supported by previous literature on online supportive communities highlighting its importance to reduce isolation and enhancing support. Further research is needed to improve the acceptability of an experience measure, including a focus on how service user stakeholders can be involved to a greater extent throughout the development process. Studies looking to replicate the structure and development of the POCEM measure in other digital contexts should consider the extent to which the development process and measure is replicable. By understanding the main outcomes and mechanisms in online mental health communities, digital healthcare providers and funders will be better placed to enable online peer support communities for mental health.

## Data Availability

The raw data supporting the conclusions of this article will be made available by the authors, without undue reservation.
