# Supplementary material for: The design and development of an experience measure for a peer community moderated forum in a digital mental health service
Source: Front Digit Health. 2022 Sep 22;4:872404. doi: 10.3389/fdgth.2022.872404 (PMC9869953; doi:10.3389/fdgth.2022.872404)
Supplement: Supplementary file 1 [file Table1.docx]

Supplementary Material

# Supplementary Tables

**Table A1: Initial pool of items and its mapping to high-level support domains, engagement type, and positive outcomes of online communities**

| Items | Engagement Type | Domain | Theme |
| --- | --- | --- | --- |
| I felt safe to share my problem | Contributor | Emo-inter | Sharing Personal Experience |
| I felt able to be myself | Reader | Emo-inter | Sharing Personal Experience |
| I didn't feel judged by others | Reader | Emo-inter | Sharing Personal Experience |
| I felt able to be honest with myself/others | Contributor | Emo-inter | Sharing Personal Experience |
| A sense of connection helped me share my personal story | Contributor | Emo-inter | Sharing Personal Experience |
| I felt able to share something personal with others | Contributor | Emo-inter | Disclosure |
| I am no longer afraid to share with others | Reader | Emo-inter | Disclosure |
| I felt comfortable seeking support from my peers | Reader | Emo-inter | Disclosure |
| I felt I could benefit from support from my peers | Reader | Emo-inter | Disclosure |
| I was able to say something for the first time today | Contributor | Emo-inter | Disclosure |
| Others helped me when I asked for help | Contributor | Emo-inter | Asking for Advice (Emotional and Informational) |
| I know who to ask for help | Reader | Emo-inter | Asking for Advice (Emotional and Informational) |
| I have influence over what this community is like | Reader | Emo-inter | Positive Modelling / Boundaries |
| Others have inspired or guided me | Reader | Emo-inter | Positive Modelling / Boundaries |
| I respect this community | Reader | Emo-inter | Positive Modelling / Boundaries |
| I feel safe in this community | Reader | Emo-inter | Positive Modelling / Boundaries |
| I felt connected | Reader | Emo-inter | Building Rapport / Socialising |
| I was able to connect with others | Reader | Emo-inter | Building Rapport / Socialising |
| I enjoyed engaging with others | Reader | Emo-inter | Building Rapport / Socialising |
| I was able to develop a meaningful relationship | Reader | Emo-inter | Building Rapport / Socialising |
| I have made new friendships | Reader | Emo-inter | Building Rapport / Socialising |
| I have felt warmth or affection for someone | Reader | Emo-inter | Building Rapport / Socialising |
| I can trust people in this community | Reader | Emo-inter | Building Rapport / Socialising |
| It was fun talking with others | Reader | Emo-inter | Building Rapport / Socialising |
| I now feel like I have helped someone | Contributor | Emo-inter | Digital Altruism |
| I now feel motivated to give advice to others | Reader | Emo-inter | Digital Altruism |
| I now feel motivated to learn more so I can help others | Reader | Emo-inter | Digital Altruism |
| I now feel able to achieve my goals | Reader | Emo-inter | Positive Reinforcement |
| I benefitted from feedback from others | Reader | Emo-inter | Positive Reinforcement |
| I now feel empowered by feedback from others | Reader | Emo-inter | Positive Reinforcement |
| I felt I was just as good as other people | Reader | Emo-inter | Perspective Shifting |
| It now feels like others believe in me | Reader | Emo-inter | Perspective Shifting |
| I am now willing to take suggestions from others | Reader | Emo-inter | Motivation for Change |
| Others have supported me to achieve my goal | Reader | Emo-inter | Evidence of Change Made |
| Sharing a personal story helped me understand my emotions | Contributor | Emo-intra | Sharing Personal Experience |
| I felt able to trust others | Contributor | Emo-intra | Disclosure |
| I felt able to ask for support | Contributor | Emo-intra | Asking for Advice (Emotional and Informational) |
| I now feel able to ask for support outside of Kooth | Reader | Emo-intra | Asking for Advice (Emotional and Informational) |
| I felt thankful for the community | Reader | Emo-intra | Gratitude |
| I now feel less alone | Reader | Emo-intra | Gratitude |
| Someone liked what I said and it made me feel good | Contributor | Emo-intra | Gratitude |
| I felt accepted | Reader | Emo-intra | Building Rapport / Socialising |
| I now feel more competent | Reader | Emo-intra | Positive Reinforcement |
| I now feel more hopeful | Reader | Emo-intra | Positive Reinforcement |
| I am now able to find solutions to my problems | Reader | Emo-intra | Positive Reinforcement |
| I can recognise the positive things I have done | Reader | Emo-intra | Perspective Shifting |
| I now believe things can improve | Reader | Emo-intra | Perspective Shifting |
| I feel optimistic for the future | Reader | Emo-intra | Perspective Shifting |
| My outlook is more positive | Reader | Emo-intra | Perspective Shifting |
| My problems now feel more managable and in perspective | Reader | Emo-intra | Perspective Shifting |
| I have been inspired to make change | Reader | Emo-intra | Motivation for Change |
| I now want to make changes in my life | Reader | Emo-intra | Motivation for Change |
| I am now hopeful for change | Reader | Emo-intra | Motivation for Change |
| I now feel excited for the future | Reader | Emo-intra | Motivation for Change |
| I am now ready to make change | Reader | Emo-intra | Motivation for Change |
| I have achieved my goal | Reader | Emo-intra | Evidence of Change Made |
| I have learned how to express myself | Contributor | Info-inter | Sharing Personal Experience |
| I have developed skills to open up to others / myself | Reader | Info-inter | Disclosure |
| I was able to use my experience to help others | Contributor | Info-inter | Positive Modelling / Boundaries |
| I think I inspired or guided others | Contributor | Info-inter | Positive Modelling / Boundaries |
| I provided information to help others | Contributor | Info-inter | Digital Altruism |
| I have learned how to support others | Reader | Info-inter | Digital Altruism |
| I have the knowledge and skills to help others | Reader | Info-inter | Digital Altruism |
| I have implemented a suggestion from someone else | Reader | Info-inter | Evidence of Change Made |
| I got information that helped me learn about myself | Reader | Info-intra | Asking for Advice (Emotional and Informational) |
| I learned something new today | Reader | Info-intra | Positive Modelling / Boundaries |
| Giving advice helped me feel positive about myself | Contributor | Info-intra | Digital Altruism |
| I now know what I need to do to feel better | Contributor | Info-intra | Positive Reinforcement |
