# Supplementary material for: The design and development of an experience measure for a peer community moderated forum in a digital mental health service
Source: Front Digit Health. 2022 Sep 22;4:872404. doi: 10.3389/fdgth.2022.872404 (PMC9869953; doi:10.3389/fdgth.2022.872404)
Supplement: Supplementary file 2 [file Table2.docx]

Supplementary Material

# Supplementary Tables

**Table A2: The pairwise comparison results for each item.**

|  |  |  |  |  |  |  |  |
| --- | --- | --- | --- | --- | --- | --- | --- |
| Item 1 | | Item 2 | | W | | p | |
| able-to-ask-for-support |  | able-to-find-solutions |  | 1.16602 |  | 1.000 |  |
| able-to-ask-for-support |  | express-myself |  | -0.12622 |  | 1.000 |  |
| able-to-ask-for-support |  | feel-safe |  | -0.75609 |  | 1.000 |  |
| able-to-ask-for-support |  | felt-accepted |  | 0.69006 |  | 1.000 |  |
| able-to-ask-for-support |  | felt-connected |  | -1.34521 |  | 1.000 |  |
| able-to-ask-for-support |  | helped-me-learn |  | 0.50868 |  | 1.000 |  |
| able-to-ask-for-support |  | helpful-to-my-problem |  | 1.46918 |  | 1.000 |  |
| able-to-ask-for-support |  | just-as-valuable |  | -0.55931 |  | 1.000 |  |
| able-to-ask-for-support |  | know-what-to-do |  | 0.25428 |  | 1.000 |  |
| able-to-ask-for-support |  | know-who-to-ask |  | 0.08608 |  | 1.000 |  |
| able-to-ask-for-support |  | learned-something |  | 0.28603 |  | 1.000 |  |
| able-to-ask-for-support |  | make-a-positive-change |  | 0.25600 |  | 1.000 |  |
| able-to-ask-for-support |  | more-hopeful |  | -0.31496 |  | 1.000 |  |
| able-to-ask-for-support |  | motivated-to-advise |  | -0.26611 |  | 1.000 |  |
| able-to-ask-for-support |  | not-judged |  | -1.60366 |  | 1.000 |  |
| able-to-ask-for-support |  | open-up-more |  | 1.52822 |  | 1.000 |  |
| able-to-ask-for-support |  | others-have-same-experiences |  | 0.03184 |  | 1.000 |  |
| able-to-ask-for-support |  | problems-more-manageable |  | 0.51798 |  | 1.000 |  |
| able-to-ask-for-support |  | skills-to-help-others |  | -0.22505 |  | 1.000 |  |
| able-to-ask-for-support |  | support-others |  | -0.72098 |  | 1.000 |  |
| able-to-ask-for-support |  | want-to-make-changes |  | 0.66100 |  | 1.000 |  |
| able-to-ask-for-support |  | No Response |  | -5.48308 |  | 0.020 |  |
| able-to-find-solutions |  | express-myself |  | -1.58618 |  | 1.000 |  |
| able-to-find-solutions |  | feel-safe |  | -2.88132 |  | 0.927 |  |
| able-to-find-solutions |  | felt-accepted |  | -0.74670 |  | 1.000 |  |
| able-to-find-solutions |  | felt-connected |  | -3.73231 |  | 0.544 |  |
| able-to-find-solutions |  | helped-me-learn |  | -0.91193 |  | 1.000 |  |
| able-to-find-solutions |  | helpful-to-my-problem |  | 0.33739 |  | 1.000 |  |
| able-to-find-solutions |  | just-as-valuable |  | -2.52674 |  | 0.982 |  |
| able-to-find-solutions |  | know-what-to-do |  | -1.18678 |  | 1.000 |  |
| able-to-find-solutions |  | know-who-to-ask |  | -1.57970 |  | 1.000 |  |
| able-to-find-solutions |  | learned-something |  | -1.13685 |  | 1.000 |  |
| able-to-find-solutions |  | make-a-positive-change |  | -1.20528 |  | 1.000 |  |
| able-to-find-solutions |  | more-hopeful |  | -2.05960 |  | 0.999 |  |
| able-to-find-solutions |  | motivated-to-advise |  | -2.16464 |  | 0.997 |  |
| able-to-find-solutions |  | not-judged |  | -4.03466 |  | 0.378 |  |
| able-to-find-solutions |  | open-up-more |  | 0.46465 |  | 1.000 |  |
| able-to-find-solutions |  | others-have-same-experiences |  | -1.54511 |  | 1.000 |  |
| able-to-find-solutions |  | problems-more-manageable |  | -0.90084 |  | 1.000 |  |
| able-to-find-solutions |  | skills-to-help-others |  | -1.83913 |  | 1.000 |  |
| able-to-find-solutions |  | support-others |  | -2.41866 |  | 0.989 |  |
| able-to-find-solutions |  | want-to-make-changes |  | -0.64918 |  | 1.000 |  |
| able-to-find-solutions |  | No Response |  | -8.88934 |  | < .001 |  |
| express-myself |  | feel-safe |  | -0.89359 |  | 1.000 |  |
| express-myself |  | felt-accepted |  | 1.11123 |  | 1.000 |  |
| express-myself |  | felt-connected |  | -1.75366 |  | 1.000 |  |
| express-myself |  | helped-me-learn |  | 0.83529 |  | 1.000 |  |
| express-myself |  | helpful-to-my-problem |  | 2.01148 |  | 0.999 |  |
| express-myself |  | just-as-valuable |  | -0.58779 |  | 1.000 |  |
| express-myself |  | know-what-to-do |  | 0.49398 |  | 1.000 |  |
| express-myself |  | know-who-to-ask |  | 0.31794 |  | 1.000 |  |
| express-myself |  | learned-something |  | 0.53171 |  | 1.000 |  |
| express-myself |  | make-a-positive-change |  | 0.51077 |  | 1.000 |  |
| express-myself |  | more-hopeful |  | -0.23233 |  | 1.000 |  |
| express-myself |  | motivated-to-advise |  | -0.17097 |  | 1.000 |  |
| express-myself |  | not-judged |  | -2.12753 |  | 0.998 |  |
| express-myself |  | open-up-more |  | 2.04741 |  | 0.999 |  |
| express-myself |  | others-have-same-experiences |  | 0.22214 |  | 1.000 |  |
| express-myself |  | problems-more-manageable |  | 0.84000 |  | 1.000 |  |
| express-myself |  | skills-to-help-others |  | -0.12269 |  | 1.000 |  |
| express-myself |  | support-others |  | -0.77151 |  | 1.000 |  |
| express-myself |  | want-to-make-changes |  | 0.98652 |  | 1.000 |  |
| express-myself |  | No Response |  | -8.27002 |  | < .001 |  |
| feel-safe |  | felt-accepted |  | 2.85871 |  | 0.932 |  |
| feel-safe |  | felt-connected |  | -1.85142 |  | 1.000 |  |
| feel-safe |  | helped-me-learn |  | 2.20691 |  | 0.997 |  |
| feel-safe |  | helpful-to-my-problem |  | 3.61437 |  | 0.611 |  |
| feel-safe |  | just-as-valuable |  | 0.48816 |  | 1.000 |  |
| feel-safe |  | know-what-to-do |  | 1.63667 |  | 1.000 |  |
| feel-safe |  | know-who-to-ask |  | 1.94825 |  | 0.999 |  |
| feel-safe |  | learned-something |  | 1.66937 |  | 1.000 |  |
| feel-safe |  | make-a-positive-change |  | 1.78461 |  | 1.000 |  |
| feel-safe |  | more-hopeful |  | 0.83311 |  | 1.000 |  |
| feel-safe |  | motivated-to-advise |  | 1.37220 |  | 1.000 |  |
| feel-safe |  | not-judged |  | -2.65356 |  | 0.968 |  |
| feel-safe |  | open-up-more |  | 3.52852 |  | 0.659 |  |
| feel-safe |  | others-have-same-experiences |  | 1.39607 |  | 1.000 |  |
| feel-safe |  | problems-more-manageable |  | 2.17005 |  | 0.997 |  |
| feel-safe |  | skills-to-help-others |  | 0.90182 |  | 1.000 |  |
| feel-safe |  | support-others |  | -0.11159 |  | 1.000 |  |
| feel-safe |  | want-to-make-changes |  | 2.18974 |  | 0.997 |  |
| feel-safe |  | No Response |  | -17.82899 |  | < .001 |  |
| felt-accepted |  | felt-connected |  | -4.01719 |  | 0.387 |  |
| felt-accepted |  | helped-me-learn |  | -0.24557 |  | 1.000 |  |
| felt-accepted |  | helpful-to-my-problem |  | 1.19318 |  | 1.000 |  |
| felt-accepted |  | just-as-valuable |  | -2.30651 |  | 0.994 |  |
| felt-accepted |  | know-what-to-do |  | -0.59789 |  | 1.000 |  |
| felt-accepted |  | know-who-to-ask |  | -1.06227 |  | 1.000 |  |
| felt-accepted |  | learned-something |  | -0.54229 |  | 1.000 |  |
| felt-accepted |  | make-a-positive-change |  | -0.62209 |  | 1.000 |  |
| felt-accepted |  | more-hopeful |  | -1.64039 |  | 1.000 |  |
| felt-accepted |  | motivated-to-advise |  | -1.83259 |  | 1.000 |  |
| felt-accepted |  | not-judged |  | -4.43828 |  | 0.201 |  |
| felt-accepted |  | open-up-more |  | 1.28604 |  | 1.000 |  |
| felt-accepted |  | others-have-same-experiences |  | -1.00872 |  | 1.000 |  |
| felt-accepted |  | problems-more-manageable |  | -0.23217 |  | 1.000 |  |
| felt-accepted |  | skills-to-help-others |  | -1.41032 |  | 1.000 |  |
| felt-accepted |  | support-others |  | -2.12116 |  | 0.998 |  |
| felt-accepted |  | want-to-make-changes |  | 0.02168 |  | 1.000 |  |
| felt-accepted |  | No Response |  | -11.86547 |  | < .001 |  |
| felt-connected |  | helped-me-learn |  | 3.21637 |  | 0.814 |  |
| felt-connected |  | helpful-to-my-problem |  | 4.55426 |  | 0.163 |  |
| felt-connected |  | just-as-valuable |  | 1.99035 |  | 0.999 |  |
| felt-connected |  | know-what-to-do |  | 2.57180 |  | 0.978 |  |
| felt-connected |  | know-who-to-ask |  | 3.30176 |  | 0.775 |  |
| felt-connected |  | learned-something |  | 2.59158 |  | 0.975 |  |
| felt-connected |  | make-a-positive-change |  | 2.79915 |  | 0.945 |  |
| felt-connected |  | more-hopeful |  | 1.97314 |  | 0.999 |  |
| felt-connected |  | motivated-to-advise |  | 3.01166 |  | 0.890 |  |
| felt-connected |  | not-judged |  | -0.92667 |  | 1.000 |  |
| felt-connected |  | open-up-more |  | 4.40141 |  | 0.215 |  |
| felt-connected |  | others-have-same-experiences |  | 2.43668 |  | 0.988 |  |
| felt-connected |  | problems-more-manageable |  | 3.15825 |  | 0.838 |  |
| felt-connected |  | skills-to-help-others |  | 1.94294 |  | 0.999 |  |
| felt-connected |  | support-others |  | 0.83304 |  | 1.000 |  |
| felt-connected |  | want-to-make-changes |  | 3.07149 |  | 0.871 |  |
| felt-connected |  | No Response |  | -16.81621 |  | < .001 |  |
| helped-me-learn |  | helpful-to-my-problem |  | 1.33819 |  | 1.000 |  |
| helped-me-learn |  | just-as-valuable |  | -1.78138 |  | 1.000 |  |
| helped-me-learn |  | know-what-to-do |  | -0.34066 |  | 1.000 |  |
| helped-me-learn |  | know-who-to-ask |  | -0.69511 |  | 1.000 |  |
| helped-me-learn |  | learned-something |  | -0.29129 |  | 1.000 |  |
| helped-me-learn |  | make-a-positive-change |  | -0.34912 |  | 1.000 |  |
| helped-me-learn |  | more-hopeful |  | -1.25917 |  | 1.000 |  |
| helped-me-learn |  | motivated-to-advise |  | -1.33910 |  | 1.000 |  |
| helped-me-learn |  | not-judged |  | -3.60596 |  | 0.616 |  |
| helped-me-learn |  | open-up-more |  | 1.41937 |  | 1.000 |  |
| helped-me-learn |  | others-have-same-experiences |  | -0.70063 |  | 1.000 |  |
| helped-me-learn |  | problems-more-manageable |  | 0.01002 |  | 1.000 |  |
| helped-me-learn |  | skills-to-help-others |  | -1.06817 |  | 1.000 |  |
| helped-me-learn |  | support-others |  | -1.74735 |  | 1.000 |  |
| helped-me-learn |  | want-to-make-changes |  | 0.23104 |  | 1.000 |  |
| helped-me-learn |  | No Response |  | -10.07349 |  | < .001 |  |
| helpful-to-my-problem |  | just-as-valuable |  | -3.17560 |  | 0.831 |  |
| helpful-to-my-problem |  | know-what-to-do |  | -1.61051 |  | 1.000 |  |
| helpful-to-my-problem |  | know-who-to-ask |  | -2.12085 |  | 0.998 |  |
| helpful-to-my-problem |  | learned-something |  | -1.55401 |  | 1.000 |  |
| helpful-to-my-problem |  | make-a-positive-change |  | -1.64990 |  | 1.000 |  |
| helpful-to-my-problem |  | more-hopeful |  | -2.59450 |  | 0.975 |  |
| helpful-to-my-problem |  | motivated-to-advise |  | -2.80196 |  | 0.944 |  |
| helpful-to-my-problem |  | not-judged |  | -4.86597 |  | 0.087 |  |
| helpful-to-my-problem |  | open-up-more |  | 0.15018 |  | 1.000 |  |
| helpful-to-my-problem |  | others-have-same-experiences |  | -2.01963 |  | 0.999 |  |
| helpful-to-my-problem |  | problems-more-manageable |  | -1.32203 |  | 1.000 |  |
| helpful-to-my-problem |  | skills-to-help-others |  | -2.33370 |  | 0.993 |  |
| helpful-to-my-problem |  | support-others |  | -2.92344 |  | 0.916 |  |
| helpful-to-my-problem |  | want-to-make-changes |  | -1.02833 |  | 1.000 |  |
| helpful-to-my-problem |  | No Response |  | -10.19715 |  | < .001 |  |
| just-as-valuable |  | know-what-to-do |  | 1.27342 |  | 1.000 |  |
| just-as-valuable |  | know-who-to-ask |  | 1.34460 |  | 1.000 |  |
| just-as-valuable |  | learned-something |  | 1.31081 |  | 1.000 |  |
| just-as-valuable |  | make-a-positive-change |  | 1.36788 |  | 1.000 |  |
| just-as-valuable |  | more-hopeful |  | 0.42951 |  | 1.000 |  |
| just-as-valuable |  | motivated-to-advise |  | 0.71488 |  | 1.000 |  |
| just-as-valuable |  | not-judged |  | -2.64273 |  | 0.970 |  |
| just-as-valuable |  | open-up-more |  | 3.13018 |  | 0.849 |  |
| just-as-valuable |  | others-have-same-experiences |  | 0.99689 |  | 1.000 |  |
| just-as-valuable |  | problems-more-manageable |  | 1.76171 |  | 1.000 |  |
| just-as-valuable |  | skills-to-help-others |  | 0.52530 |  | 1.000 |  |
| just-as-valuable |  | support-others |  | -0.39563 |  | 1.000 |  |
| just-as-valuable |  | want-to-make-changes |  | 1.83688 |  | 1.000 |  |
| just-as-valuable |  | No Response |  | -14.12888 |  | < .001 |  |
| know-what-to-do |  | know-who-to-ask |  | -0.27376 |  | 1.000 |  |
| know-what-to-do |  | learned-something |  | 0.04441 |  | 1.000 |  |
| know-what-to-do |  | make-a-positive-change |  | 0.00319 |  | 1.000 |  |
| know-what-to-do |  | more-hopeful |  | -0.83337 |  | 1.000 |  |
| know-what-to-do |  | motivated-to-advise |  | -0.84381 |  | 1.000 |  |
| know-what-to-do |  | not-judged |  | -2.95201 |  | 0.908 |  |
| know-what-to-do |  | open-up-more |  | 1.67402 |  | 1.000 |  |
| know-what-to-do |  | others-have-same-experiences |  | -0.32167 |  | 1.000 |  |
| know-what-to-do |  | problems-more-manageable |  | 0.34836 |  | 1.000 |  |
| know-what-to-do |  | skills-to-help-others |  | -0.67692 |  | 1.000 |  |
| know-what-to-do |  | support-others |  | -1.34395 |  | 1.000 |  |
| know-what-to-do |  | want-to-make-changes |  | 0.53854 |  | 1.000 |  |
| know-what-to-do |  | No Response |  | -9.11000 |  | < .001 |  |
| know-who-to-ask |  | learned-something |  | 0.32209 |  | 1.000 |  |
| know-who-to-ask |  | make-a-positive-change |  | 0.29525 |  | 1.000 |  |
| know-who-to-ask |  | more-hopeful |  | -0.70965 |  | 1.000 |  |
| know-who-to-ask |  | motivated-to-advise |  | -0.76770 |  | 1.000 |  |
| know-who-to-ask |  | not-judged |  | -3.82667 |  | 0.491 |  |
| know-who-to-ask |  | open-up-more |  | 2.14876 |  | 0.998 |  |
| know-who-to-ask |  | others-have-same-experiences |  | -0.08747 |  | 1.000 |  |
| know-who-to-ask |  | problems-more-manageable |  | 0.69530 |  | 1.000 |  |
| know-who-to-ask |  | skills-to-help-others |  | -0.53206 |  | 1.000 |  |
| know-who-to-ask |  | support-others |  | -1.33435 |  | 1.000 |  |
| know-who-to-ask |  | want-to-make-changes |  | 0.87227 |  | 1.000 |  |
| know-who-to-ask |  | No Response |  | -13.58795 |  | < .001 |  |
| learned-something |  | make-a-positive-change |  | -0.04250 |  | 1.000 |  |
| learned-something |  | more-hopeful |  | -0.87398 |  | 1.000 |  |
| learned-something |  | motivated-to-advise |  | -0.88773 |  | 1.000 |  |
| learned-something |  | not-judged |  | -2.96517 |  | 0.904 |  |
| learned-something |  | open-up-more |  | 1.62042 |  | 1.000 |  |
| learned-something |  | others-have-same-experiences |  | -0.36575 |  | 1.000 |  |
| learned-something |  | problems-more-manageable |  | 0.29928 |  | 1.000 |  |
| learned-something |  | skills-to-help-others |  | -0.71619 |  | 1.000 |  |
| learned-something |  | support-others |  | -1.37534 |  | 1.000 |  |
| learned-something |  | want-to-make-changes |  | 0.49146 |  | 1.000 |  |
| learned-something |  | No Response |  | -9.01125 |  | < .001 |  |
| make-a-positive-change |  | more-hopeful |  | -0.87486 |  | 1.000 |  |
| make-a-positive-change |  | motivated-to-advise |  | -0.91358 |  | 1.000 |  |
| make-a-positive-change |  | not-judged |  | -3.20399 |  | 0.819 |  |
| make-a-positive-change |  | open-up-more |  | 1.70865 |  | 1.000 |  |
| make-a-positive-change |  | others-have-same-experiences |  | -0.33727 |  | 1.000 |  |
| make-a-positive-change |  | problems-more-manageable |  | 0.35592 |  | 1.000 |  |
| make-a-positive-change |  | skills-to-help-others |  | -0.71009 |  | 1.000 |  |
| make-a-positive-change |  | support-others |  | -1.39635 |  | 1.000 |  |
| make-a-positive-change |  | want-to-make-changes |  | 0.54733 |  | 1.000 |  |
| make-a-positive-change |  | No Response |  | -10.16984 |  | < .001 |  |
| more-hopeful |  | motivated-to-advise |  | 0.11720 |  | 1.000 |  |
| more-hopeful |  | not-judged |  | -2.46997 |  | 0.986 |  |
| more-hopeful |  | open-up-more |  | 2.59859 |  | 0.975 |  |
| more-hopeful |  | others-have-same-experiences |  | 0.53605 |  | 1.000 |  |
| more-hopeful |  | problems-more-manageable |  | 1.25693 |  | 1.000 |  |
| more-hopeful |  | skills-to-help-others |  | 0.11474 |  | 1.000 |  |
| more-hopeful |  | support-others |  | -0.68037 |  | 1.000 |  |
| more-hopeful |  | want-to-make-changes |  | 1.38848 |  | 1.000 |  |
| more-hopeful |  | No Response |  | -10.56530 |  | < .001 |  |
| motivated-to-advise |  | not-judged |  | -3.66487 |  | 0.582 |  |
| motivated-to-advise |  | open-up-more |  | 2.78140 |  | 0.948 |  |
| motivated-to-advise |  | others-have-same-experiences |  | 0.51426 |  | 1.000 |  |
| motivated-to-advise |  | problems-more-manageable |  | 1.32499 |  | 1.000 |  |
| motivated-to-advise |  | skills-to-help-others |  | 0.02790 |  | 1.000 |  |
| motivated-to-advise |  | support-others |  | -0.88596 |  | 1.000 |  |
| motivated-to-advise |  | want-to-make-changes |  | 1.44418 |  | 1.000 |  |
| motivated-to-advise |  | No Response |  | -16.01813 |  | < .001 |  |
| not-judged |  | open-up-more |  | 4.68844 |  | 0.126 |  |
| not-judged |  | others-have-same-experiences |  | 2.86984 |  | 0.929 |  |
| not-judged |  | problems-more-manageable |  | 3.54168 |  | 0.652 |  |
| not-judged |  | skills-to-help-others |  | 2.39283 |  | 0.991 |  |
| not-judged |  | support-others |  | 1.28237 |  | 1.000 |  |
| not-judged |  | want-to-make-changes |  | 3.41045 |  | 0.722 |  |
| not-judged |  | No Response |  | -15.33947 |  | < .001 |  |
| open-up-more |  | others-have-same-experiences |  | -2.06033 |  | 0.999 |  |
| open-up-more |  | problems-more-manageable |  | -1.40497 |  | 1.000 |  |
| open-up-more |  | skills-to-help-others |  | -2.34877 |  | 0.993 |  |
| open-up-more |  | support-others |  | -2.90329 |  | 0.921 |  |
| open-up-more |  | want-to-make-changes |  | -1.12335 |  | 1.000 |  |
| open-up-more |  | No Response |  | -9.66516 |  | < .001 |  |
| others-have-same-experiences |  | problems-more-manageable |  | 0.70516 |  | 1.000 |  |
| others-have-same-experiences |  | skills-to-help-others |  | -0.39008 |  | 1.000 |  |
| others-have-same-experiences |  | support-others |  | -1.11765 |  | 1.000 |  |
| others-have-same-experiences |  | want-to-make-changes |  | 0.87757 |  | 1.000 |  |
| others-have-same-experiences |  | No Response |  | -9.93819 |  | < .001 |  |
| problems-more-manageable |  | skills-to-help-others |  | -1.06749 |  | 1.000 |  |
| problems-more-manageable |  | support-others |  | -1.74347 |  | 1.000 |  |
| problems-more-manageable |  | want-to-make-changes |  | 0.22097 |  | 1.000 |  |
| problems-more-manageable |  | No Response |  | -9.76210 |  | < .001 |  |
| skills-to-help-others |  | support-others |  | -0.73298 |  | 1.000 |  |
| skills-to-help-others |  | want-to-make-changes |  | 1.20252 |  | 1.000 |  |
| skills-to-help-others |  | No Response |  | -10.02723 |  | < .001 |  |
| support-others |  | want-to-make-changes |  | 1.82525 |  | 1.000 |  |
| support-others |  | No Response |  | -8.59153 |  | < .001 |  |
| want-to-make-changes |  | No Response |  | -8.80683 |  | < .001 |  |
|  | | | | | | | |
